# Photoelectric Measurement and Sensing: New Technology and Applications

**DOI:** 10.3390/s23208584

**Published:** 2023-10-19

**Authors:** Qibo Feng, Jiakun Li, Qixin He

**Affiliations:** MoE Key Lab of Luminescence and Optical Information, Beijing Jiaotong University, No. 3 Shangyuancun, Beijing 100044, China; jkli@bjtu.edu.cn (J.L.); heqixin@bjtu.edu.cn (Q.H.)

Laser-based measurement and sensing technology has been paid more and more attention by academia and industry because of its incomparable advantages, such as high sensitivity, fast response, and no contact. Its application has penetrated various fields of scientific research and industrial production, including industrial measurements, material analysis, environmental monitoring, and more.

In industrial production, laser measurement technology can be used for precision measurement, quality control, and inspection. For example, laser interferometry can be used to measure the shape and surface quality of components, while machine vision technology can be used to measure the distance and position of objects [1,2,3]. These technologies can help improve product quality and production efficiency. In the field of material analysis, laser spectroscopy is an important tool. For instance, laser-induced breakdown spectroscopy (LIBS) can be used to analyze the elemental composition of samples, and laser-induced fluorescence spectroscopy (LIFS) can be used to analyze the chemical substances in samples [4,5,6]. Furthermore, laser measurement and sensing technologies can also be used in environmental monitoring and safety fields. For example, laser radar can be used to measure the concentration and distribution of atmospheric pollutants, while laser absorption spectroscopy technology can be used for trace gas concentration analysis [7,8,9]. In recent years, with the development of laser sources and measurement approaches, many new technologies and applications of laser measurement and sensing have appeared [10,11].

This Special Issue aims to collect original research papers and reviews on recent developments of laser measurement technologies and innovative applications. Potential topics include, but are not limited to, laser measurement and sensing, micro- and nano-photoelectric measurement, simultaneous measurement of multiple parameters, structured light measurement, online digital measurement, computational measurement, embedded photoelectric measurement, and laser spectroscopy analysis.

Geometric parameters are important basic quantities that reflect the physical properties of an object, and they are also some of the physical quantities requiring the highest measurement accuracy in modern industrial production. For example, the measurement accuracy of a modern 3-nanometer wire’s width needs to be sub-nanometer, and laser measurement has become the only choice for such measurement [12]. Length measurement is the basis of geometric measurement. Based on the combination of length and angle measurement, the shape, size, position, and attitude of the target object can be measured with high precision. In today’s highly developed information society, fast and accurate simultaneous acquisition of a variety of information is the inevitable trend of the development of future measuring instruments; laser multi-parameter simultaneous measurement is also one of the future important development trends [13]. There are 10 articles in this Special Issue, in which different laser measurement methods have been used to measure geometric parameters.

In contribution 1, an absolute-type four-degrees-of-freedom (four-DOF) grating encoder that can simultaneously measure the three-axis pose (θx, θy, θz) and one-axis out-of-plane position of an object with high accuracy was demonstrated. The presented grating encoder was composed of a stationary reading head and a movable grating reflector. The authors investigated the modeling and decoupling algorithms to guarantee an independent calculation of these four-DOF absolute positions. In addition, the authors constructed a prototype and verified that the proposed grating encoder could achieve the absolute measurement of four-DOF θx, θy, θz, and Z with an accuracy of sub-arcseconds and sub-micrometers. The encoder proposed in this research is the first one to achieve absolute simultaneous measurements of four-DOF position and pose with a large measurement range. The success of this new grating encoder can benefit various multi-DOF positioning applications, especially for large-scale synthetic aperture optics (SAO), including stitching off-axis parabolic mirrors and pulse compression grating.

In contribution 2, a precision 3D measurement instrument integrating multiple laser range sensors was designed, which fuses the information of multiple redundant laser range sensors to obtain the coordinates of a 3D position. The authors developed an identification model of laser beam position and orientation parameters based on redundant distance information and standard spherical constraint to reduce the requirement for the assembly accuracy of laser range sensors. In addition, they designed a hybrid identification algorithm of PSO-LM (particle swarm optimization Levenberg–Marquardt) to solve the high-order nonlinear problem of the identification model. Experiments of identification of position and orientation, verifications of the measuring accuracy, and calibration of industrial robots were conducted, which show the effectiveness of the proposed 3D measurement instrument and identification methods. Moreover, the proposed instrument is small and can be used in narrow industrial sites.

The authors of contribution 3 proposed an adaptive hybrid sampling method for free-form surfaces based on geodesic distance. The free-form surfaces are divided into segments, and the sum of the geodesic distance of each surface segment is taken as the global fluctuation index of free-form surfaces. The number and location of the sampling points for each free-form surface segment are reasonably distributed. Compared with the common methods, this method can significantly reduce the reconstruction error for the same sampling points. This method overcomes the shortcomings of the current commonly used method of taking curvature as the local fluctuation index of free-form surfaces, and provides a new perspective for the adaptive sampling of free-form surfaces.

Contribution 4 introduced a targetless and simultaneous measurement method of three-degrees-of-freedom (3-DOF) angular motion errors using digital speckle pattern interferometry (DSPI). Based on the analysis of the sensitivity mechanism of DSPI to DOF errors and the formation mechanism of the phase fringes, the relationship between the angular motion errors and the distribution of the interferometric phases was established, and a new simultaneous measurement model of 3-DOF angular motion errors was further proposed by the authors. Furthermore, repetitive tests, noise tests, and precision analysis were carried out to verify the performance of the system. The test results showed that the measurement resolution of the system was <1 μrad, which is capable of measuring the pitch angle, yaw angle, and roll angle at the submicron arc level simultaneously without target mirrors.

In contribution 5, an improved calibration method based on coplanar constraint was proposed for a camera with a large FOV. Firstly, with an auxiliary plane mirror provided, the positions of the calibration grid and the tilt angles of the plane mirror are changed several times to capture several mirrored calibration images. Secondly, the initial parameters of the camera are calculated based on each group of mirrored calibration images. Finally, adding the coplanar constraint between each group of calibration grids, the external parameters between the camera and the reference plane are optimized via the Levenberg–Marquardt algorithm (LM). The experimental results show that the proposed camera calibration method has good robustness and accuracy.

The authors of contribution 6 described a novel laser scattering instrument that measures mass concentration and particle size in real time over a wide concentration range. The instrument combines laser scattering and time-of-flight aerodynamics in one optical device. In this study, two APD detectors were used to receive the forward-scattered light and the side-scattered light, respectively, which can increase the sensitivity greatly. In addition, a high-speed ADC and FPGA were combined to achieve an anti-overlap algorithm objective to measure the high concentrations of aerosol. It was verified in experiments that the anti-overlapping algorithm can effectively improve the applicability of the aerodynamic particle size spectrometer under high concentration conditions.

In contribution 7, a compact and high-precision three-degrees-of-freedom (DOF; X, Y, and Z directions) grating encoder based on quadrangular frustum pyramid (QFP) prisms was proposed in this paper to solve the insufficient installation space problem of the reading head of the multi-DOF in high-precision displacement measurement applications. The authors built a three-DOF measurement platform through the self-collimation function of the miniaturized QFP prism. The overall size of the reading head is 12.3 × 7.7 × 3 cm^3^, and it has the potential for further miniaturization. The measurement accuracy of the main displacement is below 500 nm on average; the minimum and maximum errors are 0.07% and 2.84%, respectively. This design will help further popularize the research and applications of multi-DOF grating encoders in high-precision measurements.

In contribution 8, an evaluation method based on fitting planes was proposed to evaluate laser plane attitude and determine the degree of laser coplanarity effectively. Real-time fitting of laser planes with three planar targets of different heights provides information about the laser plane attitude on both sides of the rails. On this basis, laser coplanarity evaluation criteria were developed to determine whether the laser planes on both sides of the rails are coplanar. Using the method in this study, the laser plane attitude can be quantified and accurately assessed on both sides, effectively resolving the problem with traditional methods that can only assess the laser plane attitude qualitatively and roughly, thereby providing a solid foundation for calibration and error correction of the measurement system.

In contribution 9, a high-precision real-time pose measurement method for the primary lens of a space telescope in orbit based on laser ranging was proposed. The measurement of the pose of the primary lens in real time and with high precision is one of the important techniques for a space telescope. Through this method, the pose change of the telescope’s primary lens can be easily calculated through six high-precision laser distance changes. Analysis and experiments show that this method can accurately obtain the pose of the primary lens in real time. The rotation error of the measurement system is 2 × 10^−5^ degrees (0.072 arcsecs), and the translation error is 0.2 μm. This study will provide a scientific basis for high-quality imaging of a space telescope.

Contribution 10 proposed a LiDAR-based method for sensing the thickness of tunnel wet spray, which aims to improve efficiency and quality. The proposed method utilizes an adaptive point cloud standardization processing algorithm to address differing point cloud postures and missing data, and the segmented Lamé curve is employed to fit the tunnel design axis using the Gauss–Newton iteration method. This establishes a mathematical model of the tunnel section and enables the analysis and perception of the thickness of the tunnel to be wet-sprayed through comparison with the actual inner contour line and the design line of the tunnel. Experimental results show that the proposed method is effective in sensing the thickness of tunnel wet spray, with important implications for promoting intelligent wet spraying operations, improving wet spraying quality, and reducing labor costs in tunnel lining construction.

Besides geometric parameter measurement, laser technology also has a wide range of applications in vibration measurement, molecular concentration detection, and more.

The authors of contribution 11 studied the most relevant figures of merit (FoM) of a DNTT-based organic phototransistor as a function of the timing parameters of light pulses to assess the device’s suitability for real-time applications. The dynamic response to light pulse bursts at ~470 nm (close to the DNTT absorption peak) was characterized at different irradiances under various working conditions, such as pulse width and duty cycle. Several bias voltages were explored to allow for a trade-off to be made between operating points. Amplitude distortion in response to light pulse bursts was also addressed.

In contribution 12, the measurement characteristics of speckles based on the photoinduced electromotive force (photo-emf) effect for high-frequency, small-amplitude, and in-plane vibration were theoretically and experimentally studied. The relevant theoretical models were utilized. A GaAs crystal was used as the photo-emf detector for experimental research, as well as to study the influence of the amplitude and frequency of the vibration, the imaging magnification of the measuring system, and the average speckle size of the measuring light on the first harmonic of the induced photocurrent in the experiments. The correctness of the supplemented theoretical model was verified, and a theoretical and experimental basis was provided for the feasibility of using GaAs to measure in-plane vibrations with nanoscale amplitudes.

Contribution 13 introduced a small-scale water quality detection instrument that can detect two representative water quality parameters: the permanganate index and total dissolved solids (TDS). The permanganate index measured through the laser spectroscopy method can show the approximate value of organic matter in the water, and the TDS measured through the conductivity method can show the approximate value of inorganic matter in the water. In addition, the authors proposed an evaluation method of water quality based on the percent scores to facilitate the popularization of civilian applications. The instrument designed in this paper has the advantages of high sensitivity, high integration, and small volume, which lays the foundation for the popularity of the detection instrument.

The authors of contribution 14 studied a thermal management system for battery modules (BTMS) of a hybrid train. The authors analyzed the flow rates in each branch and the pressure losses. Since many branches of this system are built inside the battery box of the hybrid train, flow rate measurements were conducted by means of an ultrasonic clamp-on flow sensor because of its minimal invasiveness and its ability to be quickly installed without modifying the system layout. Experimental data of flow rate and pressure drop were then used to validate a lumped parameter model of the system, realized in the Simcenter AMESim^®^ environment.

Contribution 15 mainly focuses on the dust sensing system based on the light scattering method. The authors minimized the cost by replicating the particle count (PC) of an existing dust sensing device. The existing device uses multiple sensors to measure the number of particles according to the size of dust. In this study, the authors attempted to replicate the performance of a multi-sensor device through a single-sensor device to minimize the power consumption and reduce the cost of the dust sensing system.

Machine learning and deep learning have been readily adopted in laser measurement and sensing [14,15,16]. Using machine learning can improve the automation level and efficiency of optoelectronic detection systems. By training and optimizing machine learning algorithms with optoelectronic detection data, automatic processing and analysis of data can be achieved, reducing manual intervention time and error rates, and improving measurement accuracy and efficiency. This Special Issue includes two articles that combine optoelectronic detection with machine learning.

Contribution 16 introduced an improved blind/referenceless image spatial quality evaluator (BRISQUE) algorithm. The algorithm was formulated by using image characteristic extraction technology to obtain a characteristic vector (CV) that consisted of 36 characteristic values that could effectively reflect the defocusing condition of the corresponding image. The authors constructed an image database that contained a sufficient number of training samples. The trained model is trained to obtain the support vector machine (SVM) model by using the regression function of the SVM. The method of establishing the image definition evaluation model via SVM is feasible and yields higher subjective and objective consistency.

Contribution 17 introduced a polarization imaging device of cotton foreign fiber based on the difference in optical properties and polarization characteristics between cotton fibers. The authors proposed an object detection and classification algorithm based on an improved YOLOv5 to achieve small foreign fiber recognition and classification. In the algorithm, the lightweight network Shufflenetv2 with the Hard-Swish activation function was used as the backbone feature extraction network. The PANet network connection of YOLOv5 was modified to obtain a fine-grained feature map to improve the detection accuracy for small targets. A CA attention module was added to the YOLOv5 network to increase the weight of the useful features while suppressing the weight of invalid features to improve the detection accuracy of foreign fiber targets. The model volume, mAP@0.5, mAP@0.5:0.95, and FPS of the improved YOLOv5 were up to 0.75 MB, 96.9%, 59.9%, and 385 f/s, respectively, compared to YOLOv5, and the improved YOLOv5 increased by 1.03%, 7.13%, and 126.47%, respectively, which proves that the method can be applied to the vision system of an actual production line for cotton foreign fiber detection.

Contributions 18 and 19 are review papers. Contribution 18 focused on the wheel flat detection techniques and flat signal processing methods based on wayside deployment. The timely and accurate detection of wheel flats is of great significance to ensure the safety of train operation and reduce maintenance costs. Commonly used wheel flat detection methods, including sound-based methods, image-based methods, and stress-based methods, are introduced and summarized. The advantages and disadvantages of these methods are discussed, and conclusions are drawn. In addition, the flat signal processing methods corresponding to different wheel flat detection techniques are also summarized and discussed by the authors.

In contribution 19, the advances in research on oscillation principles and key technologies of the different kinds of dual-frequency solid-state lasers are reviewed, including birefringent dual-frequency solid-state lasers and biaxial and two-cavity dual-frequency solid-state lasers. The system composition, operating principle, and some main experimental results are briefly introduced. Several typical frequency difference stabilizing systems for dual-frequency solid-state lasers are introduced and analyzed. The main development trends of research on dual-frequency solid-state lasers are predicted.

In summary, This Special Issue presents a variety of advanced laser measurement techniques and their interesting applications in many areas. We hope that this SI will help researchers to better understand the state of the art of laser-based measurement and sensing technologies. We hope that the 19 published papers will also help researchers working in the field to disclose future perspectives.

## List of Contributions

1. Wang, S.; Luo, L.; Zhu, J.; Shi, N.; Li, X. An Ultra-Precision Absolute-Type Multi-Degree-of-Freedom Grating Encoder. *Sensors* **2022**, *22*, 9047; https://doi.org/10.3390/s22239047.
2. Gao, G.; Kuang, L.; Liu, F.; Xing, Y.; Shi, Q. Modeling and Parameter Identification of a 3D Measurement System Based on Redundant Laser Range Sensors for Industrial Robots. *Sensors* **2023**, *23*, 1913; https://doi.org/10.3390/s23041913.
3. Chen, C.; Jia, H.; Lu, Y.; Zhang, X.; Chen, H.; Yu, L. An Adaptive Hybrid Sampling Method for Free-Form Surfaces Based on Geodesic Distance. *Sensors* **2023**, *23*, 3224; https://doi.org/10.3390/s23063224.
4. Shi, L.; Wu, S.; Yan, M.; Niu, H. A Targetless Method for Simultaneously Measuring Three-Degree-of-Freedom Angular Mo-tion Errors with Digital Speckle Pattern Interferometry. *Sensors* **2023**, *23*, 3393; https://doi.org/10.3390/s23073393.
5. Lu, R.; Wang, Z.; Zou, Z. Accurate Calibration of a Large Field of View Camera with Coplanar Constraint for Large-Scale Specular Three-Dimensional Profile Measurement. *Sensors* **2023**, *23*, 3464; https://doi.org/10.3390/s23073464.
6. Zhang, J.; Zhang, Z.; Hou, L.; Zhou, W. A Novel Optical Instrument for Measuring Mass Concentration and Particle Size in Real Time. *Sensors* **2023**, *23*, 3616; https://doi.org/10.3390/s23073616.
7. Wang, S.; Liao, B.; Shi, N.; Li, X. A Compact and High-Precision Three-Degree-of-Freedom Grating Encoder Based on a Quadrangular Frustum Pyramid Prism. *Sensors* **2023**, *23*, 4022; https://doi.org/10.3390/s23084022.
8. Wang, L.; Wang, H.; Han, Q.; Fang, Y.; Wang, S.; Wang, N.; Li, G.; Ren, S. A Laser Plane Attitude Evaluation Method for Rail Profile Measurement Sensors. *Sensors* **2023**, *23*, 4586; https://doi.org/10.3390/s23104586.
9. Shi, H.; Du, J.; Wang, L.; Bian, J.; Gao, G.; Liu, D.; Fan, B.; Yang, H. A High-Precision Real-Time Pose Measurement Method for the Primary Lens of Large Aperture Space Telescope Based on Laser Ranging. *Sensors* **2023**, *23*, 4833; https://doi.org/10.3390/s23104833.
10. Xu, D.; Song, Q.; Fang, S.; Guo, Y. Sensing Method for Wet Spraying Process of Tunnel Wall Based on the Laser LiDAR in Complex Environment. *Sensors* **2023**, *23*, 5167; https://doi.org/10.3390/s23115167.
11. Campajola, M.; Di Meo, P.; Di Capua, F.; Branchini, P.; Aloisio, A. Dynamic Photoresponse of a DNTT Organic Phototran-sistor. *Sensors* **2023**, *23*, 2386; https://doi.org/10.3390/s23052386.
12. Gao, J.; Zhang, B.; Feng, Q.; Shen, X.; Xue, Y.; Liu, J. Speckle Measurement for Small In-Plane Vibration Using GaAs. *Sensors* **2023**, *23*, 2724; https://doi.org/10.3390/s23052724.
13. Tian, Z.; Chen, H.; Ding, Q.; Che, X.; Bi, Z.; Wang, L. Research on Small-Scale Detection Instrument for Drinking Water Combined Laser Spectroscopy and Conductivity Technology. *Sensors* **2023**, *23*, 2985; https://doi.org/10.3390/s23062985.
14. De Rosa, R.; Romagnuolo, L.; Frosina, E.; Belli, L.; Senatore, A. Validation of a Lumped Parameter Model of the Battery Thermal Management System of a Hybrid Train by Means of Ultrasonic Clamp-On Flow Sensor Measurements and Hydronic Optimization. *Sensors* **2023**, *23*, 390; https://doi.org/10.3390/s23010390.
15. Lee, S.; Kwon, J.; Park, D. Optimized Replication of ADC-Based Particle Counting Algorithm with Reconfigurable Multi-Variables in Pseudo-Supervised Digital Twining of Reference Dust Sensor Systems. *Sensors* **2023**, *23*, 5557; https://doi.org/10.3390/s23125557.
16. Zhang, N.; Lin, C. The Image Definition Assessment of Optoelectronic Tracking Equipment Based on the BRISQUE Algo-rithm with Gaussian Weights. *Sensors* **2023**, *23*, 1621; https://doi.org/10.3390/s23031621.
17. Wang, R.; Zhang, Z.; Yang, B.; Xi, H.; Zhai, Y.; Zhang, R.; Geng, L.; Chen, Z.; Yang, K. Detection and Classification of Cotton Foreign Fibers Based on Polarization Imaging and Improved YOLOv5. *Sensors* **2023**, *23*, 4415; https://doi.org/10.3390/s23094415.
18. Fu, W.; He, Q.; Feng, Q.; Li, J.; Zheng, F.; Zhang, B. Recent Advances in Wayside Railway Wheel Flat Detection Techniques: A Review. *Sensors* **2023**, *23*, 3916; https://doi.org/10.3390/s23083916.
19. Jiao, M.; Jiang, F.; Xing, J.; Liu, Y.; Lian, T.; Liu, J.; Li, G. Advances of Research on Dual-Frequency Solid-State Lasers for Synthetic-Wave Absolute-Distance Interferometry. *Sensors* **2023**, *23*, 3206; https://doi.org/10.3390/s23063206.
